# Antigenic and functional profiles of a *Lawsonia intracellularis* protein that shows a flagellin-like trait and its immuno-stimulatory assessment

**DOI:** 10.1186/s13567-018-0515-0

**Published:** 2018-02-15

**Authors:** Gayeon Won, John Hwa Lee

**Affiliations:** College of Veterinary Medicine, Chonbuk National University, Iksan Campus, Gobong-ro 79, Iksan, 54596 Republic of Korea

## Abstract

The obligate intracellular *Lawsonia intracellularis* (LI), the etiological agent of proliferative enteropathy (PE), is an economically important disease in the swine industry. Due to extreme difficulty of in vitro culture of the pathogen, molecular characterization of protein components of LI that are targets of the immune system, is difficult; thus, the scientific evidence to drive the development of preventive measures is lacking. In this work, we investigated the antigenic and functional characteristics of a putative flagellar-associated protein, LI0570, using in silico computational approaches for epitope prediction and an in vitro protein-based molecular assay. The amino acid sequence of LI0570 exhibited similarities to flagellar-associated proteins in four different bacterial strains. The presence of B cell linear confirmative epitopes of the protein predicted by a bioinformatics tool was validated by western blot analysis using anti-LI mouse hyperimmune serum, which implied that LI0570 induced production of antigen-specific antibodies in vivo. Further, TLR5-stimulating activity and IL-8 cytokine expression produced via downstream signaling were observed in HEK-Blue™-hTLR5 cells stimulated with LI0570. This result indicates that the LI0570 protein can trigger an innate immune response followed by a T-cell-related adaptive immune response in an infected host. Collectively, the data presented here support that the LI0570 protein which shows the antigenic potential could be a useful component of a recombinant vaccine against PE, providing progress toward an effective prevention strategy.

## Introduction

Porcine proliferative enteropathy (PE) is caused by *Lawsonia intracellularis* (LI), an obligate intracellular bacterium, commonly occurring in swine herds. The infection results in thickening of the intestinal epithelium due to enterocyte proliferation. Clinical features such as chronic enteritis, lethargy, retarded growth rate, diarrhea, and acute hemorrhagic enteritis causing sudden death are exhibited. Indeed, Porcine PE is responsible for severe economic loss in the swine industry worldwide [1]. Proliferative enteropathy (PE) has also been diagnosed in a variety of animals such as horses, rabbits, rats, guinea pigs, dogs, chickens, sheep, deer, and non-human primates [2]. Despite high herd prevalence of LI infection in growing-finishing pigs, its pathogenic mechanisms remain speculative due to difficulty of in vitro culture of this obligate intracellular bacterium.

Intracellular motility of LI ultimately leads to cell dissemination, which enhances LI’s ability to penetrate mucous layers and colonize the intestinal tract. Other enteroinvasive bacteria pathogens such as *Listeria* and *Shigella* are randomly detected in the intestinal cell cytoplasm [3], while LI is largely present in the apical region of enterocytes [1]. Thus, molecular mechanisms of actin-based motility by which other intracellular bacterial pathogen spread in the infected cells may differ from those adapted by LI [4]. Interestingly, some enteric bacterial pathogens have mechanisms to penetrate the mucus layer to reach epithelial cells via flagella-driven motility which plays a role in the initial phase of infection [5, 6]. The presence of a single flagellar motor on LI, which is one of the main phenotypic elements of the pathogen, was observed in the supernatant of an infected cell culture in vitro [7] and also a preliminary analysis of the LI genome sequence (PHE/MN1-00; NCBI accession #NC_008011) exhibits genes responsible for flagellar assembly. It is also known that the bacterial protein flagellin effectively induces an innate immune response of the host that is mediated by its ability to bind to toll-like receptor 5 (TLR5). Flagellin, acting via TLR5, leads to activation of MyD88-dependent signaling and the proinflammatory transcription factor, NF-κB, which induces an intense innate and adaptive immune response against flagellated bacteria [8]. Although other bacterial flagellin has been widely used as an adjuvant molecule and as an antigen in vaccinology [9], the ability of LI flagellin to trigger the activation of immuno-modulatory pathway has not been demonstrated.

In this work, we initiated the study on the functional characteristics of LI0570 annotated by the United States National Center for Biotechnology Information (NCBI) as a putative flagellin and related hook-associated protein and its antigenic traits. We putatively defined LI0570 as Lawsonia flagellin (LFliC). Antigenic characteristics of LFliC were assessed by bioinformatics tools for in silico B-cell prediction. Further, we investigated whether the LI0570 retained immuno-adjuvant characteristics. To elucidate the role of the TLR5 agonist, TLR5-stimulating activity and IL-8 expression by the purified flagellin protein were measured using HEK293 cells.

## Materials and methods

### Bioinformatics analysis

Sequence analysis of the *Lawsonia* flagellin protein LI0570 was performed using the NCBI BLAST programs [10] and FASTA program [11]. Sequence similarity analysis was assessed by creating multiple sequence alignments using CLUSTALW [12]. The antigenicity index (linear B-cell epitopes) for the LFliC protein was estimated in silico using the BepiPred 2.0 web server [13]. BepiPred 2.0 employs the hidden Markov model combined with amino acid propensity scales to predict epitope data derived from crystal structures by assessing surface accessibility, helix probability, sheet probability, and coil probability [14].

### Construction of the protein expression system

The predicted nucleotide sequences corresponding to amino acid sequences of LFliC (LI0570) were chemically synthesized (Bioneer, Daejeon, Korea). The synthesized genes were digested with *Eco*RI and *Hin*dIII endonucleases and subcloned into the *Escherichia coli* expression vector pET28a (+), which introduced a 6×His tag at the N-terminus of expressed proteins. The constructed plasmids pET28a-*LfliC* were transformed into *E. coli* BL21 (DE3) pLysS cells by electroporation and designated JOL1682. The His-tagged recombinant protein was purified from culture supernatant of JOL1682 strain expressing *LfliC* according to an affinity purification procedure using a nickel-charged nitrilotriacetic acid (Ni–NTA) resin as previously described [15]. Subsequently, the protein was quantified by Bradford assay. The bacterial strains and plasmids applied in this study are listed in Table 1.

**Table 1:** **Bacterial strains and plasmids used in this study**

Strains/plasmids	Description	References
Strains		
BL21(DE3)pLysS	*F*-*ompT hsdSB (rB*- *mB*-*) dcm galλ (DE3) pLysS Cmr*	Promega
JOL1682	*E. coli* BL21(DE3) pLysS expressing *LfliC* via pET28a (+) system	This study
Plasmids		
pET28(+)	IPTG-inducible, T7 expression vector, C-terminal 6×His tag, Kan^R^	Novagen, USA
pET28(+)-*LfliC*	pET28a derivative containing *LfliC*	This study

### Immunoblotting

Western blot analysis was performed to evaluate immunoreactivity of the LFliC protein in vitro using anti-LI hyperimmune serum obtained from the mice immunized with an *L*. *intracellularis* modified-live vaccine (Enterisol^®^ Ileitis, Boehringer Ingelheim, Ingelheim am Rhein, Germany). The mice were inoculated with 5 × 10^6.9^ 50% tissue culture infectious doses (TCID50) via an oral route twice at 2-week intervals. The purified protein (10 μg/μL) was subjected to 15% SDS-PAGE and then transferred onto a polyvinylidene difluoride (PVDF) membrane. The membrane was blocked with phosphate-buffered saline (PBS) buffer containing 3% bovine serum albumin (BSA) for 1 h, followed by incubation with the anti-LI mouse hyperimmune serum at a 1:300 dilution for 2 h. The antigen–antibody interaction was detected with a 1:5000 diluted horseradish peroxidase (HRP)-conjugated goat anti-mouse IgG (Southern Biotechnology, Birmingham, AL, USA; diluted 1:5000).

### In vitro characterization of LFliC protein

Determination of TLR5 bioactivity with the putative *Lawsonia* flagellin was conducted using HEK-Blue™-hTLR5 cells (InvivoGen, San Diego, CA, USA). The cells were incubated in Dulbecco’s modified Eagle’s medium (DMEM) supplemented with 10% fetal bovine serum (FBS), zeocin (50 μg/mL), and blasticidin (1 μg/mL). Cells (1 × 10^6^ cells per well) that had been incubated overnight in a 96-well plates were stimulated with the LFliC protein (10 μg) in duplicate for various time periods (0, 1, 3, 4, and 7 h). The expression of TLR5 on the stimulated cells was assessed by fluorescence-activated cell sorting (FACS) analysis using an anti-TLR5 monoclonal antibody (1 μg/mL; Anti-hTLR5-IgG2a, Thermo Fisher Scientific, Waltham, MA, USA) as previously described [16]. Furthermore, expression of IL-8 cytokine produced by signaling through TLR5 was measured in the stimulated cells by reverse transcription-polymerase chain reaction (RT-PCR). The primers for the IL-8 gene were obtained previously [17]. Briefly, 1 × 10^6^ HEK cells were treated with two concentrations of LFliC (10 and 100 ng/mL) in duplicate. The treated cells were incubated at 37 °C in a 5% CO_2_ incubator for 24 h and the transcription level of IL-8 was determined by RT-PCR as described previously [18].

### In vitro cytotoxicity assay

The cytotoxic potential of the LFliC protein was assessed by cell viability assay with thiazolyl blue tetrazolium bromide (3-(4,5-dimethylthiazol-2-yl)-2,5-diphenyltetrazolium bromide; MTT) [19]. Cells from a rat intestinal epithelial cell line (IEC-18) were plated at a concentration of 1 × 10^6^ and were stimulated with various concentrations of LFliC (0.1, 1, 5, and 10 ng/mL) in duplicate. The pulsed cells were incubated at 37 °C in a 5% CO_2_ incubator for 24 h, and then the cells were incubated for an additional 4 h with 5 mg/mL MTT. The bromide salt of MTT was absorbed by the cells and was reduced to a product called formazan within the mitochondria, which accumulated in the cell. The resulting blue formazan crystals was solubilized by dimethyl sulfoxide (DMSO), and the absorbance value was measured at 560 nm using a microplate spectrophotometer.

### Statistical analysis

Non-parametric Mann–Whitney test was used to evaluate the statistical difference using GraphPad Prism (GraphPad Software Inc., La Jolla, CA, USA). *P*-values less than 0.05 were considered statistically significant.

## Results

### Characterization of LFliC as a putative antigen

**Figure 1:**
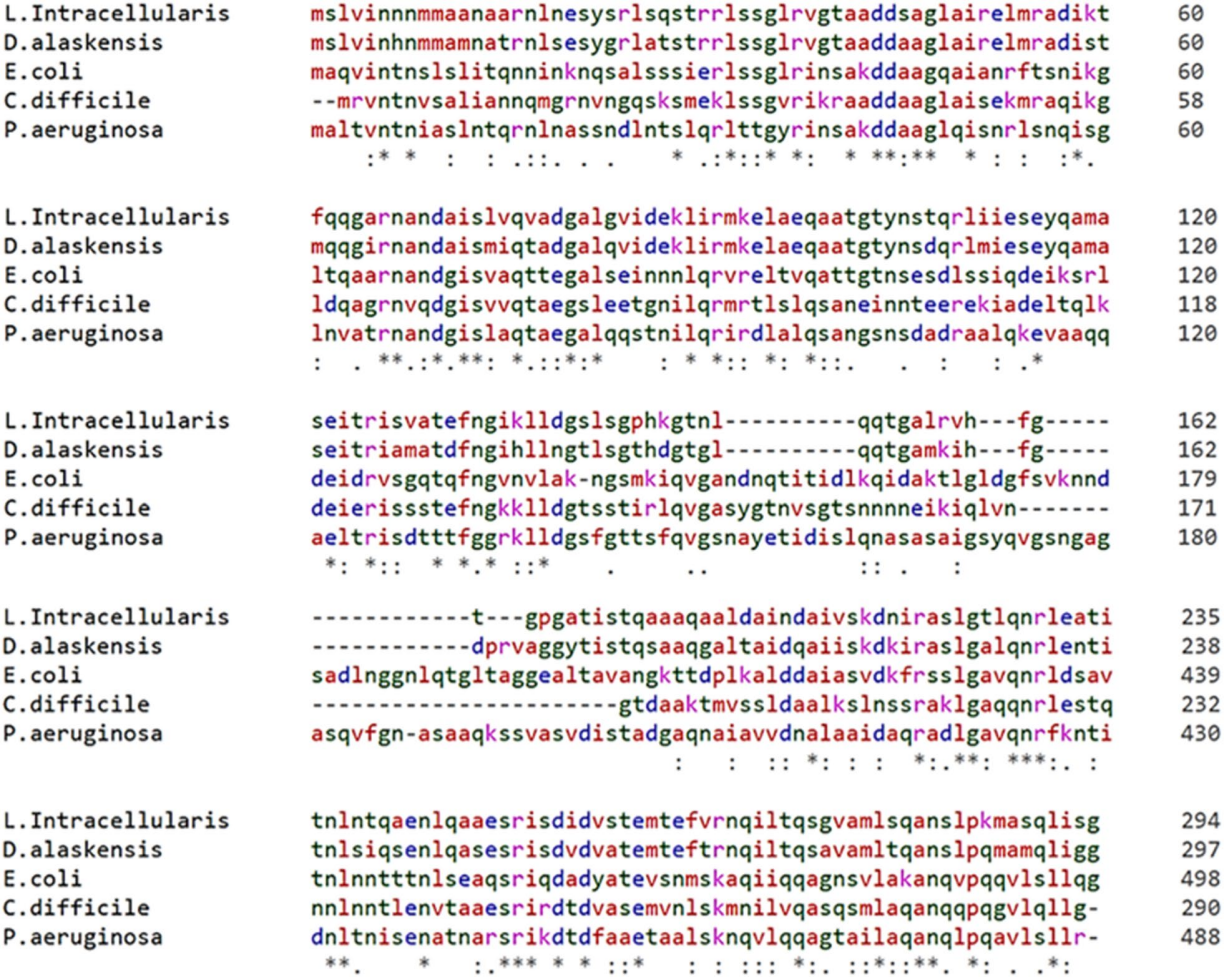
**Sequence alignments of LI0570 and other flagellin-associated proteins using the CLUSTALW alignment program.** An asterisk (*) indicates the amino acid residues that are identical in all sequences in the alignment, a colon (:) indicates conserved substitutions, and a period (.) indicates semi-conserved substitutions.

Conserved domains of the LFliC (LI0570) protein in the *L. intracellularis* strain (PHE/MN1-00) were analyzed by searching NCBI’s Conserved Domain Database (CDD). The analysis showed that the protein contained conserved regions of flagellin and a related hook-associated protein FlgL (accession ID: COG1344; E-value, 4.38e−49) between amino acids 16–292 of the protein, a bacterial flagellin N-terminal helical region (accession ID: pfam00669; E-value, 2.40e−23) between amino acids 16–141 and a flagellar hook-associated protein (accession ID: TIGR02550; E-value, 7.74e−16) between amino acids 16–273. BLASTP similarity searches also revealed that the full amino acid sequence of LFliC (LI0570) of *L. intracellularis* strain (PHE/MN1-00) showed substantial similarity to the flagellin protein of *Desulfovibrio alaskensis* (Sequence ID: WP_011367650.1), *Clostridium difficile* (Sequence ID: YP_001086707.1), *Pseudomonas aeruginosa* (Sequence ID: NP_249783.1), and *E. coli* K-12 (Sequence ID: NP_416433.1) (Table 2). Sequence features of the multiple sequence alignment of LFliC to the flagellin-associated proteins of the four individual bacterial strains are shown in Figure 1. The identity varied among the strains at the protein levels (Table 2), with regions of extensive similarity between amino acids 1–151 and 202–294 of the LFliC sequence (Figure 1). This in silico characterization and analysis indicated that a putative *Lawsonia* flagellin protein, LI0570, shares a large degree of full-length amino acid sequence homology (40–77% identical residues) to the defined proteins as flagellin in other bacterial organisms (Table 2).

**Table 2:** **Homologues of LI0570 in the amino acid sequence of flagellin associated protein searched by the BlastP program**

Protein/strain	Max score	Total score	Query cover (%)	E-value	Identity	Accession
Flagellin/*Desulfovibrio alaskensis*	451	451	100	2e−163	77	WP_011367650.1
Flagellar filament structural protein/*Escherichia coli* str. K-12 substr. MG1655	108	174	76	2e−25	44	NP_416433.1
B-type flagellin/*Pseudomonas aeruginosa* PAO1	112	187	95	6e−27	40	NP_249783.1
Flagellin C/*Clostridioides difficile* 630	167	167	99	6e−49	40	YP_001086707.1

### In vitro and in vivo characterization of immunogenic proteins

**Figure 2:**
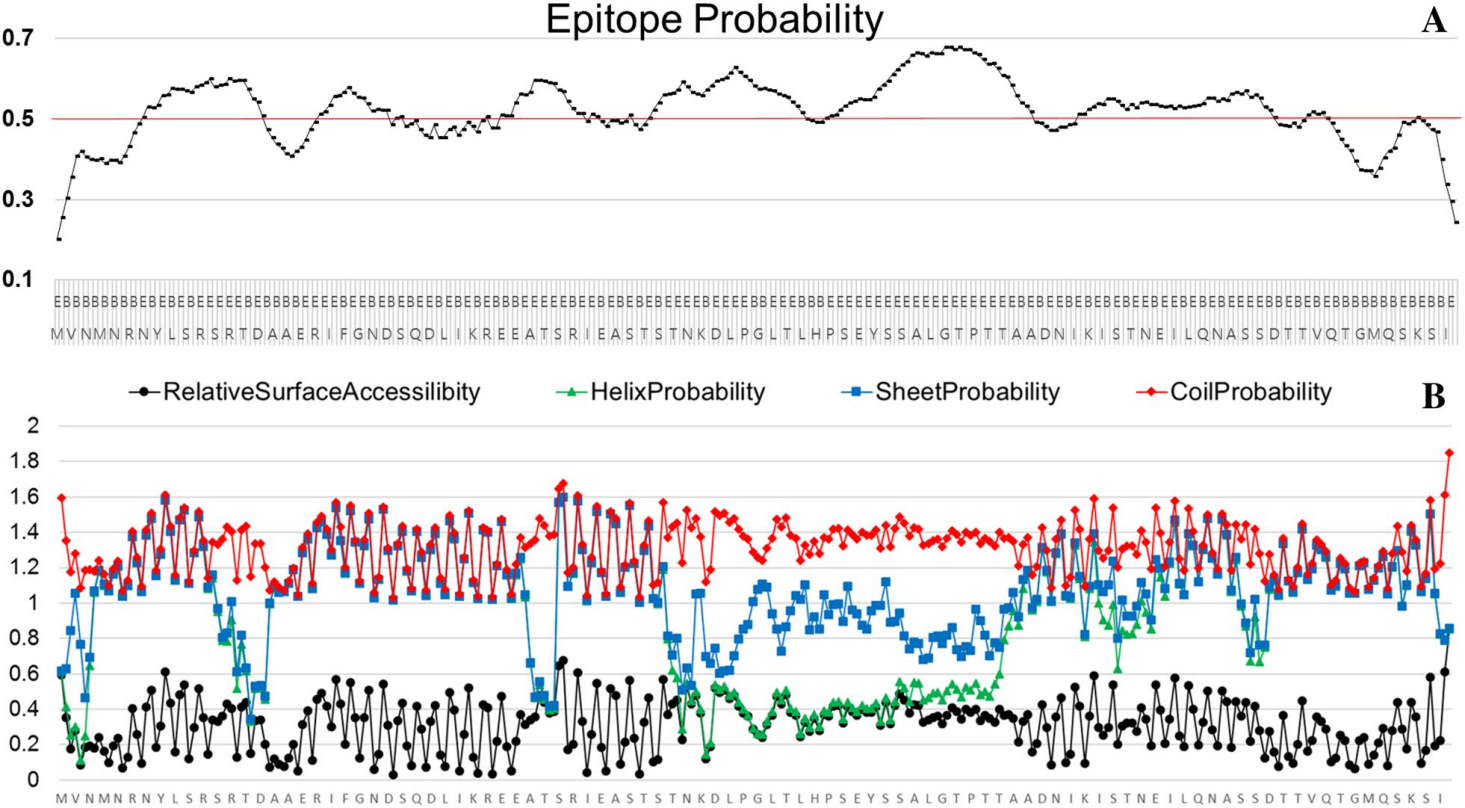
**Computational approach to predict conserved B-cell epitopes in the LI0570 protein sequence. A** Epitope probability prediction plot of the protein LI0570. **B** Prediction of B-cell epitopes by different propensity scales. The BepiPred 2.0 program was used for linear epitope prediction of the LFliC protein. The cut-off value (0.5) was automatically determined by the BepiPred 2.0 program.

**Figure 3:**
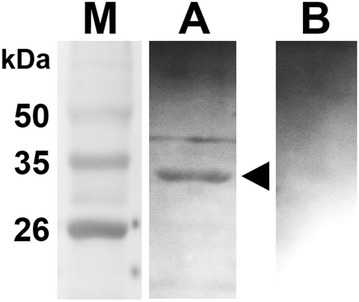
**Western blot analysis of purified LFliC.** The protein was strongly recognized by mouse hyperimmune serum obtained from a mouse immunized with *L. intracellularis.* The arrow in lane A indicates a ~ 33 kDa band, which is the predicted size of LFliC fused with 6×His. Serum from an unimmunized mouse was used as a negative control (lane B). Lane M, size marker.

To evaluate potential antigenic features of the LI0570 protein, potential linear B-cell epitopes of the protein recognized as antigens in a highly selective manner were predicted using the BepiPred 2.0 program. Overall epitope probability was determined based on the value of the propensity scales, and the probability value of a specific residue greater than a user-defined threshold (by default 0.5) was considered for B-cell linear epitopes (Figure 2). These variable domains might represent unique epitopes. The surface probability plot indicates that most of the amino acids were surface exposed. Further, to assess in vivo antigenic characteristics of the LFliC protein, western blot analysis using mouse anti-LI hyperimmune serum was performed. The serum identified a distinct band near 33 kDa, which shows that mouse anti-LI immune serum had intensive immunoreactivity with the LFliC protein (Figure 3). The presence of antibodies specific to a particular antigen protein indicates that immune response against the protein could be induced in the infected host.

### LFliC characterization

**Figure 4:**
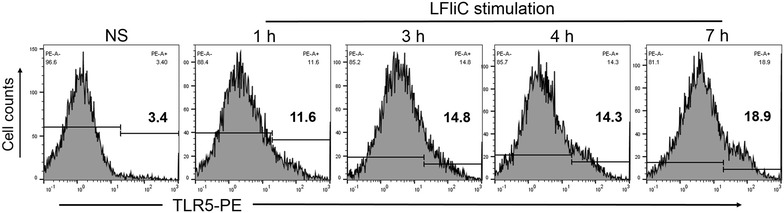
**Functional characterization of LI0570 protein.** Representative FACS histogram of the surface marker-positive cell population. TLR5 expression was measured in HEK-Blue™-hTLR5 cells stimulated with 100 ng/mL of LFliC for various time periods (1, 3, 4, and 7 h). Data shown represent a histogram of samples stained with PE-labeled anti-hTLR5 antibody.

**Figure 5:**
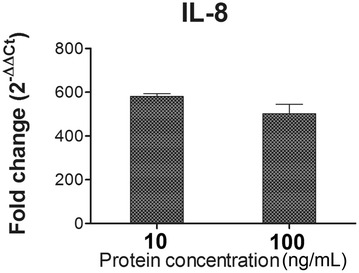
**IL-8 chemokine assay in LFliC pulsed cells.** Relative quantification (2^−∆∆Ct^) of IL-8 mRNA level in HEK-Blue™-hTLR5 cells treated with 10 and 100 ng/mL of LFliC in duplicate. Data represent the mean ± standard deviation (s.d.).

Flagellin, a TLR5 agonist, is a potent activator of the innate immune system. To evaluate the immune stimulatory capacity of LFliC protein, bioactivities of TLR5 and IL-8 were assessed in the presence of purified LI flagellin protein using HEK-Blue™-hTLR5 cells stimulated with the flagellin protein. Increased TLR5 surface expression was detected between 1 and 7 h post-infection in cells stimulated with LFliC (Figure 4). We observed an approximate sixfold increase in the surface expression of TLR5 on cells following 7 h of stimulation compared with that on untreated cells (Figure 4). IL-8 mRNA was also upregulated in pulsed cells (Figure 5). The stimulation led to approximate 590- and 515-fold increases in IL-8 mRNA level in cells treated with LFliC protein at concentrations of 10 and 100 ng/mL, respectively (Figure 5).

### In vitro cytotoxicity assay

**Figure 6:**
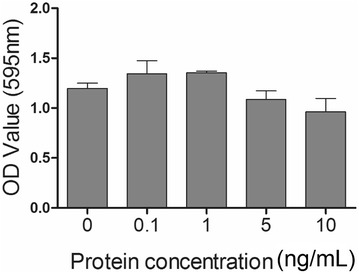
**MTT viability assay to assess LhlyA cytotoxicity.** In vitro cytotoxicity of various concentrations of LFliC in IEC-18 cells was determined using the MTT assay in duplicate. Data represent the mean ± standard deviation (s.d.).

In vitro cytotoxicity of the LFliC protein was assessed in the IEC-18 cell line by MTT colorimetric assay following exposure of the LFliC protein for 24 h. After stimulation with the cells at various concentrations of the protein, salt formazan derivatives produced by the mitochondria activity were estimated by absorbance at 560 nm. No significant difference in absorbance value was observed in the cells treated with concentrations of 0.1, 1, 5, and 10 ng/mL LFliC protein (Figure 6). Considering the selective ability of living cells to reduce 3-[4, 5-dimethyl-thiazol-2-yl]-2, 5 diphenyl tetrazolium bromide (MTT) into formazan [19], the rate of conversion to formazan was associated with the metabolic activity of the cells. This result shows that the target protein might not have affected the deteriorative viability of the intestinal cells.

## Discussion

Porcine PE caused by *L*. *intracellularis* is a common intestinal disease affecting susceptible pigs and poses a substantial economic loss to swine industries. Due to the obligate intracellular life style of the pathogen, conventional laboratory approaches to reveal molecular genetic traits such as antibiotic resistance have been restricted, and development of prevention strategies has been hampered [20]. Despite the completion of LI genome sequencing, protein immunogens against LI infection have yet to be characterized. Proteomic and comparative transcriptional analyses were recently conducted to identify functions related to the pathogenic mechanisms of the LI proteins [7, 21].

In this study, we predicted functional properties and the antigenic potential of the putative LI flagellin protein LI0570. By BLASTP analysis, the amino acid sequence of LI0570 displayed a certain degree of similarity to the amino acid sequence of a flagellin-associated protein in *D. alaskensis*, *E. coli* K-12, *P. aeruginosa*, and *C. difficile.* Particularly, the B-type flagellin of *P. aeruginosa* showed 40% similarity of sequence identity to LI0570 and partially contributed to effective production of a protective antibody against *P. aeruginosa* infection [22]. Immunotherapy with IgG antibodies specific to the N-terminus of the B-type flagellin protein also resulted in effective reduction of mortality and morbidity in a pathogen-infected murine burn model [23]. Given that LI0570 showed substantial similarity to the N-terminal region of *P. aeruginosa* (Figure 1), the putative LFliC could hypothetically be considered a target for the host immune system.

In this work, the capacity of LFliC as a potential immunogen was confirmed by in silico analysis for linear B-cell epitope prediction and in vitro immunoblotting (Figures 2, 3). Given that an antigen–antibody interaction is fundamental to inducing a humoral immune response to invading pathogens [24], prediction of B-cell epitopes is necessary to evaluate its potential antigenic property. To elucidate whether the predicted B-cell epitope within the LFliC protein reflected its actual antigenicity, western blot analysis was performed using mouse hyperimmune serum raised against LI infection (Figure 3). An immuno-reactive band corresponding to the predicted size of the LFliC protein (33 kDa) was recognized with mouse polyclonal antibodies against LI, which indicated that the LFliC protein may possess potential antigenic determinants. However, the epitope recognition by the mouse anti-LI serum may not guarantee the recognition by a swine convalescent serum, which is still needed to be confirmed in the further study.

Bacterial flagellin, a pathogen-associated molecular pattern (PAMP), binds to TLR5 on the surface of dendritic cells (DC) or epithelial cells [25] and directly or indirectly induces innate and adaptive immune responses [26]. The D1 and D2 domains of bacterial flagellin, two highly conserved regions, are crucial for the recognition of TLR5 [27]. Following activation with the TLR5 ligand flagellin, TLR5-dependent secretion of IL-8, which is controlled by activation of the transcription factor NF-κB and MAPK, necessitates stimulating a signal transduction cascade via the TLR5 pathway [17]. In this study, LFliC protein effectively induced enhanced expression of TLR5 on HEK cells (Figure 4). Further, the transcriptional level of chemokine IL-8 mRNA, which is a TLR5-mediated proinflammatory gene, also increased in the stimulated HEK cells (Figure 5). The results imply that LI0570 possesses intrinsic immuno-stimulatory characteristics involved in inducing innate immunity. Further, cell-mediated immunity is an important feature involved in host defense against intracellular bacterial infection [28]. Given that previous studies reported that bacteria flagellin functions as stimulators for differentiation of CD4^+^ T cell via DC activation [8], more work is needed to understand the involvement of the LFliC protein in T-cell immunity mediated by DC.

Taken together, the results presented suggest that the LI0570 protein annotated by NCBI as a putative flagellin and related hook-associated protein of LI shows immuno-antigenicity in silico and in vitro, providing evidence that the protein could produce antigen-specific antibodies in vivo. Further, the capacity of LI0570 to induce increased expression of TLR5 suggests that the prominent antigens trigger innate immunity and prime antigen-specific adaptive immunity. Currently, a licensed avirulent live vaccine strain against LI is commercially available and has the potential for reactogenicity [29]. The structural and functional findings of LI0570 in this study could aid in the production of safe recombinant vaccines, and these results will serve as an important groundwork for improving the prevention of PE more generally.
